# Approaches to estimating inbreeding coefficients in clinical isolates of *Plasmodium falciparum* from genomic sequence data

**DOI:** 10.1186/s12936-016-1531-z

**Published:** 2016-09-15

**Authors:** John D. O’Brien, Lucas Amenga-Etego, Ruiqi Li

**Affiliations:** 1 Department of Mathematics, Bowdoin College, 8600 College Station, Brunswick, ME USA; 2 Wellcome Trust Centre for Human Genetics, University of Oxford, Roosevelt Drive, Oxford, UK; 3 Navrongo Health Research Centre, Upper East Region, Navrongo, Ghana

**Keywords:** Inbreeding coefficient, MOI, COI, F-statistics, Balding–Nichols model

## Abstract

**Background:**

The advent of whole-genome sequencing has generated increased interest in modelling the structure of strain mixture within clinical infections of *Plasmodium falciparum* The life cycle of the parasite implies that the mixture of multiple strains within an infected individual is related to the out-crossing rate across populations, making methods for measuring this process in situ central to understanding the genetic epidemiology of the disease.

**Results:**

This paper derives a set of new estimators for inferring inbreeding coefficients using whole genome sequence read count data from *P. falciparum* clinical samples, which provides resources to assess within-sample mixture that connect to extensive literatures in population genetics and conservation ecology. Features of the *P. falciparum* genome mean that standard methods for inbreeding coefficients and related *F*-statistics cannot be used directly. After reviewing an initial effort to estimate the inbreeding coefficient within clinical isolates of *P. falciparum*, several generalizations using both frequentist and Bayesian approaches are provided. A simpler, more intuitive frequentist estimator is shown to have nearly identical properties to the initial estimator both in simulation and in real data sets. The Bayesian approach connects these estimates to the Balding–Nichols model, a mainstay within genetic epidemiology, and a possible framework for more complex modelling. A simulation study shows strong performance for all estimators with as few as ten variants. Application to samples from the PF3K data set indicate significant across-country variation in within-sample mixture. Finally, a comparison with results from a recent mixture model for within-sample strain mixture show that inbreeding coefficients provide a strong proxy for these more complex models.

**Conclusions:**

This paper provides a set of methods for estimating inbreeding coefficients within *P. falciparum* samples from whole-genome sequence data, supported by simulation studies and empirical examples. It includes a substantially simple estimator with similar statistical properties to the estimator in current use. These methods will also be applicable to other species with similar life-cycles. Implementations of the methods described are available in an open-source R package pfmix. Estimates for the PF3K public data release are provide as part of this resource.

## Background

While genetic factors play a crucial role in the emergence of drug resistance within *Plasmodium falciparum*, many aspects of the genetic epidemiology of the parasite remain obscure [1, 2]. The beginnings of a global perspective on the genetic structure of parasite populations emerged from the analysis of whole-genome sequencing data (WGS) derived from ~200 parasite genomes collected directly from clinical patients in six countries on three continents [3]. This study gave further evidence for the widespread presence of within-isolate strain mixture and significant amounts of variation in its degree across continents. In grappling with the complexity of WGS read count data, the study departed from standard approaches for quantifing the amount of within-sample variation by instead using an inbreeding coefficient, $f_{ws}$, a form of F-statistic.

Strain mixture has been traditionally assessed via multiplicity of infection (MOI) [4–6], using methods for inferring the number of strains from single-nucleotide polymorphisms (SNPs) or other typing technologies applied at a small number of loci. Researchers have subsequently shown how finite mixture models can infer MOI using WGS but the under the heading of complexity of infection (COI) as these models can capture additional mixture features [7, 8]. Still, inbreeding coefficients have a long connection to population genetics and conservation biology and may be of interest to researchers connecting *P. falciparum* studies to other genetic contexts [9, 10]. This paper presents a collection of statistical methods for estimating $f_{ws}$, explores their performance in simulation, details their connection to COI estimates, and confirms the variation in $f_{ws}$ values across countries using the *P. falciparum* 3000 genomes (PF3K), a publicly available data resource.

Inbreeding coefficients and the F-statistics from which they derive are measurements of the departure of allelic heterozygosity observed within a population from those expected at Hardy–Weinberg equilibrium (HWE) [10, 11]. HWE specifies the distribution of alleles assuming panmixia, a population exhibiting perfectly random mating with an absence of mutation, migration, drift, selection or other effects [12]. F-statistics calibrate the empirical allele distribution within a subpopulation against those expected under HWE, ranging from a value of one (no mixture) to zero (perfect HWE-type mixture). In the context of comparing the parasites’ genetic diversity within a single infected individual relative to the local geographic population (and absent any geographic structuring of the population, i.e. the Wahlund effect), these statistics effectively become inbreeding coefficients. $f_{ws}$ denotes the relative amount of inbreeding within an individual sample (*w*) relative to the expected amount in a subpopulation (*s*). Since here estimates are only considered only relative to a single country (subpopulation), the use of paired subscripts, $f_{ws}$ is deprecated in favour of $f_i$ for a specific sample *i*.

F-statistics have proven to be an effective and extremely popular means for investigating species’ population structure from both allelic and genomic data [10, 13, 14]. However, standard software tools assume specific ploidy structures incommensurate with WGS data from *P. falciparum* and so cannot be used directly. The critical difference is that, within a human host, *P. falciparum* exists only in the haploid stage of its life-cycle [15]. Since short-read WGS data cannot yet capture full haplotypes, individual reads cannot be uniquely identified with their strain of origin. Without being able to associate reads to individual *P. falciparum* strains, no ’out-of-the-box’ use of standard *F*-statistics approaches with this new data appears possible.

Several earlier works have applied the F-statistic framework to *P. falciparum* within-sample mixture. These concepts—while not under the heading of inbreeding coefficients—undergird much of the seminal work on MOI estimation [5, 6]. More recently, Manske et al. [3] provided an initial estimator for inbreeding coefficients using WGS based on the slope of a modified regression line between the expected and observed heterozygosity within a sample. Auburn et al. [16] explores the connection between this estimator and standard MOI approaches by comparing these estimates with MOI values inferred by genotyping the *msp-1* and *msp-2* genes, showing strong correlation between these values in their sample sets.

This estimator has been further utilized in a number of recent studies on *P. falciparum*, including analyses of populations in the Gambia, Ghana, and Guinea [17, 18]. It has also been used in analysis of the population structures of *Plasmodium vivax* and *Plasmodium knowlesi* [19, 20]. A recent examination of this estimator in the context of microsatellite genotyping explores a strong relationship between the number of variants, allele frequency, and estimator performance[21]. There has been otherwise little statistical work characterizing this estimator or it’s properties. This paper seeks to remedy some of this deficit by providing: a simple presentation of this estimator; a set of alternate estimators that make stronger connections to the tradition around *F*-statistics; an investigation of their properties through simulations; and several applications to relevant data sets.

This paper proceeds as follows. First, an overview of the data and the notation is provided. The initial estimator employed by Manske et al. [3] for estimating $f_i$ is then reviewed, followed by the presentation of two additional frequentist estimators. A Bayesian approach for these statistics is then derived from the the Balding–Nichols model. All of these estimators are compared in an extensive simulation study. To consider their empirical performance, the correlation across all estimators in 344 Ghanaian samples is examined and the Bayesian estimates are compared to COI estimates. To show the performance under controlled circumstances, we apply the methods to several clonal laboratory strains. As a final example, the estimates for the PF3K sample set are presented for each country, confirming significant variation in the amount of within-sample mixture across countries. The conclusion provides brief discussion of the strengths and limitations of the approaches, and possible future directions for modelling within-sample mixture using WGS.

## Data and models

### Data and preparation

The data used comes largely from Release 3.0 of the PF3K resource. An overview of this project, collection protocols, and a full sequencing protocol can be found at the consortial website [22]. For all the samples considered below, data come from Illumina HiSeq sequencing applied to clinical *P. falciparum* samples collected from 14 countries. Starting from the publicly available vcf files, samples from Nigeria and Senegal were also excluded due to sample size and differing sequencing technology, respectively. First, only positions that exhibited minor allele frequencies greater than 0.01 were retained. Variants were furthered filtered at the country level by removing samples that exhibited fewer than 80 % of variant positions with at least 20× coverage. SNPs with less than 20× coverage were then removed from all remaining samples. This yielded variable number of SNPs within countries, from 1108 in Cambodia to 6596 in Laos. The number of samples within each country ranged from 35 for Laos to 344 in Ghana. Four additional samples—two replicates each of DD2 and 7G8—were taken from Release 5.0 of the PF3K resource for use as negative control. Each of these samples comprised a single, unmixed strain and was sequenced to high coverage (~65×). These were cleaned according to the steps above, yielding 23,109 viable positions. A subset of 1000 SNPs were randomly selected of those remaining for inference here.

### Notation

Within a country, samples are indexed $i = 1, \ldots , N$ and the SNPs by $j=1,\ldots ,M$. At SNP *j* within sample *i*, we observe $r_{ij}$ reads that agree with the reference, and $n_{ij}$ reads that are different from the reference. $p_{ij}$ denotes the allele frequency for reference allele for SNP *j* in sample *i* and estimate it via the maximum-likelihood estimator (MLE) for proportions: $\hat{p}_{ij} = \frac{r_{ij}}{r_{ij} + n_{ij}}$. Similarly, $p_j$ denotes the population-level reference allele frequency for each SNP and is estimated according to the across-sample MLE:

1 $$\begin{aligned} \widehat{p}_{j}\,=\, & {} \sum _{i=1}^N n_{ij} \bigg / \sum _{i=1}^N (r_{ij} \,+\, n_{ij}). \end{aligned}$$

All MLEs are calculated by country. Table 1 is provided as a reference to the reader for notation.

**Table 1:** Notation for parameters used throughout the manuscript

Parameter	Description
$j \,=\, 1,\ldots , M$	Index over number of SNPs, *M*
$i\,=\, 1,\ldots , N$	Index over number of samples, *N*
$r_{ij}, \ n_{ij}$	Reference/non-reference read count data in sample *i* at variant *j*
$d_{ij}\,=\, (r_{ij},n_{ij})$	Read count data in sample *i* at variant *j*
$p_j$ ($\widehat{p}_j$)	Population-level non-reference allele frequency for SNP *j* (estimate)
$p_{ij}$,$(\widehat{p}_{ij})$	Within-sample non-reference allele frequency for SNP j in sample i (estimate)
$f_i$	Inbreeding coefficient for sample *i*
$H_o(b,i)$	Observed heterozygosity for sample *i* in bin *b*
$H_e(b)$	Expected heterozygosity for bin *b*
$\widehat{f}^{*}_i$	Estimator of $f_i$ by method $*$.
$\ {f}, \ {p}$	Vector of $f_i$ and $p_j$’s in Bayesian model

### A previous frequentist estimator for $f_i$, and two alternatives

In Manske et al., the authors provide an initial approach to estimating $f_i$. This estimator is referred to as $f_i^{(m)}$ to contrast it with subsequent estimators. For each sample *i*, the estimator first partitions alleles into 10 equally-spaced bins based on their minor allele frequency: $(0,\,0.05),\ldots ,\, (0.45,\,0.50)$ . Within each bin, *b*, the average expected heterozygosity assuming country-level HWE is calculated by

2 $$\begin{aligned} H_e\,(b)\,=\, & {} \frac{1}{M_b}\sum _{k \in b}^{M_b} 2 \cdot \widehat{p}_k \cdot (1 - \widehat{p}_k) , \end{aligned}$$

where $M_b$ is the number of SNPs within bin *b*. The average observed heterozygosity within each bin and each sample is calculated by

3 $$\begin{aligned} H_o(b,i)= & {} \frac{1}{M_b}\sum _{k \in b}^{M_b} 2 \cdot \widehat{p}_{ik} \cdot (1 - \widehat{p}_{ik}) \text{. } \end{aligned}$$

Finally, $\hat{f}_i^{(m)}$ is calculated as $1-\beta$ where $\beta$ is the slope found by regressing the $H_o(b,\,i)$ values against $H^e_{b}$ values centered within their respective allele frequency bins and constrained to pass through the origin. This is the initial estimator.

The binning procedure, while stabilizing the estimator against influence from an excess of low frequency alleles common within samples, may also introduce bias. This effect can be removed by discarding the binning procedure in favour of directly regressing observed heterozygosity for each SNP against the expected value, still constrained to pass through the origin. This provides a closed-form expression for the regressed estimator, $f_i^{(r)}$, as

4 $$\begin{aligned} \widehat{f}_i^{(r)} \,=\, 1-\frac{\displaystyle \sum \nolimits _{j=1}^M \widehat{p}_j \cdot (1-\widehat{p}_j) \cdot \widehat{p}_{ij} \cdot (1-\widehat{p}_{ij}) }{ \displaystyle \sum \nolimits _{j=1}^M \widehat{p}_j^2 \cdot (1-\widehat{p}_j)^2} \text{. } \end{aligned}$$

A similar estimator, more transparently connected to the ideas underpinning traditional *F*-statistics, can be found in the following way. For a single SNP *j*, suppose $f_i$ to be the fraction of the population-level heterozygosity equal to the difference between the population-level heterozygosity, $H_j^p$ and the sample-level heterozygosity, $H_j^i$ that is,

5 $$\begin{aligned} f_i \cdot H_j^p = H_j^p - H_j^i. \end{aligned}$$

Dividing through by $H_j^p$ gives an estimate for $f_i$ for the SNP *j*. Averaging across all SNPs and taking the ratio of expectations to be the expectation of the ratios gives the estimator

6 $$\begin{aligned} \widehat{f}^{(d)}_i\,=\, & {} 1- \frac{\displaystyle \sum \nolimits _{j=1}^M \widehat{p}_{ij} \,(1-\widehat{p}_{ij})}{ \displaystyle \sum \nolimits _{j=1}^M \widehat{p}_{j} \,(1-\widehat{p}_{j})}. \end{aligned}$$

This is the direct estimator, since it contains the critical ratio of the mean observed heterozygosity over the mean expected heterozygosity characteristic of F-statistics.

For each of these estimators, a bootstrap approach is employed to estimate the variance in the estimates for confidence intervals [23, 24]. The bootstrap works by assuming that the empirically observed distribution – here, the allele frequencies – provides a reasonable approximation to the true empirical distribution. By repeatedly subsampling with replacement from the observed distribution and recalculating the estimator at each iteration, a distribution of estimates is built from which confidence intervals can be calculated.

### Bayesian model framework

Inbreeding coefficients comparable to the above estimators can also be derived by the Balding—Nichols model, a widely used method for measuring inbreeding in other genetic contexts [25]. This approach also has strong similarities to previous work in the context of *P. falciparum* [5, 6]. In using this model, several simplifying assumptions are required. SNPs are treated as unlinked (i.e. no linkage disequilibrium) and it is assumed that individual parasites within a sample represent a random sample of the surrounding population. It is further assumed that read counts are sampled identically, independently, and represent an unbiased sample of allele frequency $p_{ij}$.

#### Likelihood and priors

The approach for the Bayesian estimator adapts the Balding–Nichols model of allele frequency within inbred subpopulations to the specific context of *P. falciparum* WGS data [25, 26]. In *P. falciparum* the relevant subpopulation is the collection of parasites within a clinical sample. For sample *i* and SNP *j*, conditional upon an inbreeding coefficient $f_i$ and a population-level allele frequency $p_j$, the Balding-Nichols model gives the allele frequency $p_{ij}$ as a Beta distribution:

7 $$\begin{aligned} p_{ij}\sim & {} \mathcal {B}\,\bigg (\frac{1-f_i}{f_i}p_j,\,\frac{1-f_i}{f_i}\,(1-p_j)\bigg ). \end{aligned}$$

Since the read counts are assumed to be identical and independent, $p_{ij}$ is drawn from a Beta distribution, and the probability of the data is binomial, the conjugacy of these distributions can be used to eliminate the dependence on the unknown $p_{ij}$ and give a Beta-binomial distribution for the likelihood at a site *j* and position *j*:

8 $$\begin{aligned} \mathbb {P}(r_{ij},n_{ij}| p_j, f_i)\,=\, & {} {r_{ij} + n_{ij} \atopwithdelims ()n_{ij} } \frac{\text{ B }\left(n_{ij} + \frac{1-f_i}{f_i} p_j, r_{ij} + \frac{1-f_i}{f_i}(1-p_j)\right)}{\text{ B }\left(\frac{1-f_i}{f_i} p_j,\frac{1-f_i}{f_i} (1-p_j)\right)} , \end{aligned}$$

where $\text{ B }(\ \cdot \ , \ \cdot )$ is the beta function. Since independence by site and by sample is assumed, the complete likelihood of the data, $\mathcal {D}$ conditional upon the inbreeding coefficients for all samples within the population, $\mathbf {f} = (f_1, \cdots , f_N)$ and the allele frequency for all SNPs $\mathbf {p} = (p_1,\cdots , p_M)$ becomes

9 $$\begin{aligned} \mathbb {P}(\mathcal {D}|\mathbf {f}, \mathbf {p}) \,=\, \displaystyle \prod _{i=1}^N \prod _{j=1}^M \mathbb {P}(r_{ij},\,r_{ij}|f_i,\,p_j). \end{aligned}$$

The only prior information about the $f_i$ values suggests that high levels of inbreeding are common but not obligatory in west African populations, and this is quantitatively interpreted as a uniform prior on each $f_i$ between zero and one. Similarly, a uniform prior distribution is put on each allele frequency, although rare variants were eliminated as part of data cleaning described in the Data preparation subsection above.

#### Inference

Since the posterior distribution is not known in closed form, standard random-walk Metropolis-Hastings Markov chain (MCMC) approach is used to numerically approximate it [27, 28]. The Metropolis-Hastings algorithm constructs a discrete-time Markov chain over the parameter space in such a way that the posterior distribution of the chain is the stationary distribution of the chain. This requires that at a given iteration in the chain, the move from the current parameter state *x* to new parameter state $x'$ with probability $\alpha$ occurs according to

10 $$\begin{aligned} \alpha\,=\, & {} \text{ min }\left ( \frac{\mathbb {P}(x'|\mathcal {D})}{\mathbb {P}(x|\mathcal {D})}\cdot \frac{ \mathbb {P}(x' \rightarrow x) }{ \mathbb {P}(x \rightarrow x') },\,1 \right ) \,=\, \text{ min } ( \alpha _1 \cdot \alpha _2, \ 1 ). \end{aligned}$$

The first ratio is that the posterior probabilities of *x* and $x'$, and written as $\alpha _1$. The second ratio, $\alpha _2$, gives probability of choosing the current state from the proposed state over the reverse move. Since $\alpha _1$ constitutes assessment of the likelihood and the prior functions that can be calculated directly from the specifications above, only the calculation for $\alpha _2$ is shown. Proposed parameters are denoted with an apostrophe.

- $\mathbf {f}$ —randomly select *i* and propose $f_i$ from $\mathcal {B}(\alpha _{c_i},\beta _{c_i})$, leading to $\alpha _2 = \frac{\mathcal {B}(f_i|\alpha _{c_i},\beta _{c_i})}{\mathcal {B}(f_i'|\alpha _{c_i},\beta _{c_i})}$.
- $\mathbf {p}$ —randomly select *j* and then draw the proposed parameter $p_j$ from the uniform prior, leading to $\alpha _2 = 1$.
- $\varvec{\alpha },\ \varvec{\beta }$ —for both of these parameters, randomly select individual components and propose new values directly from the prior distribution, leading to $\alpha _2 = \frac{\exp {(-x)}}{\exp {(-x')}}$ where *x* and $x'$ are the current and proposed state of the relevant component.

The autocorrelation of the log-posterior has minimal lag. As a secondary check, the chain was run for all of the chromosomes individually and compared values with the complete data set. Since SNPs are treated as independent, the performance of the model should be unaffected if the model performs similarly across chromosomes. Across all chromosomes performance is nearly identical, with greater than $95\,\%$ correlation among maximum *a posteriori* (MAP) estimates.

### Implementation

All code was implemented in the R computational environment [29]. The set of scripts implementing each of the estimators, the MCMC algorithm, visualizations, data simulations, and filtered data sets are available at the pacakge website [30]. This repository includes a tutorial and workflow for completing analyses using these approaches. All materials are released under a creative commons license.

## Results

### Simulations

To compare the qualities of the four estimators, a simulation study was performed under a range of parameter values to capture how estimator performance may vary with the quality of data collected in the field. The number of SNPs, the number of read counts at each SNP, the degree of skew in the allele frequency distribution ($\beta$, described below), and the amount of inbreeding were examined. For each parameter set, 100 replicate data sets were simulated. The full set of parameters are listed in Table 2. For comparing these results to empirical data, it is important to note that the coverage level is more comparable to the the minimum coverage level, rather than the average coverage level which can vary substantially. This is because, absent other errors, the coverage level determines the statistical properties of the within-sample allele frequency, with the standard error of the estimate scaling inversely with the square root of the minimum coverage.

**Table 2:** Parameter values for simulated data sets

Parameter	Description	Simulation values
M	Number of SNPs	10, 50, 150, 500, 1500
C	Total read counts per SNP	10, 100, 1000, 10000
f	Inbreeding coefficient	0.01, 0.1, 0.5, 0.9, 0.99
$\beta$	Controls skew in allele frequency	1, 10, 100, 1000

For each parameter set, 100 replicate data sets were generated

The simulated data were created by first fixing the inbreeding coefficient *f* and the allele frequency distribution. The skewness of the underlying allele frequency was parameterized as a Beta distribution with parameters $\alpha$ and $\beta$. $\alpha$ was fixed to one, while $\beta$ was varied according to the simulation to induce differening degrees of skew. As $\beta$ increases, the distribution becomes increasingly right-skewed: when $\beta =1$ then 1 % of alleles have less than a 0.01 frequency while when $\beta =1000$ more than 99 % of allele have less than a 0.01 frequency. For a fixed $\beta$ and *f*, *M* alleles are then sampled from the Beta distribution with parameters defined by Eq. 7. The read counts were then simulated according to a binomial distirbution with those within-sample allele frequencies.

**Fig. 1:**
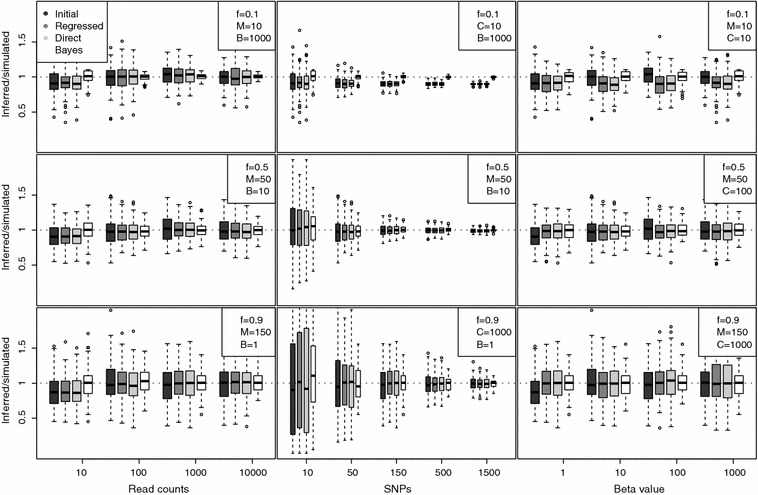
Inferred value over simulated values for each estimator across a range of parameter values: $M\,=\,10\,-\,1500$, $C\,=\,10\,-\,10,000$, $f\,=\,0.01\,-\,0.99$, and $\beta\,=\,1\,-\,1000.$ Vertical axis shows inferred/simulated value, with dashed line at one. Specific simulated values can be found in Table 2. Each Tukey boxplot represents 100 replicate data sets with the same parameters

Figure 1 summarizes the comparison of $f_i$ point estimates made by the initial, regressed, direct, and Bayesian estimators across the simulated data. All boxplots use Tukey’s design, showing median, inter-quartile range and whiskers up to 1.5 times the inter-quartile range. Inferred/simulated values are reported as a measure of performance. Across all parameter values, the estimators performed similarly, with the Bayesian estimate showing the least bias and highest accuracy. The number of SNPs proves the largest determinant of performance, with 50 SNPs sufficient to ensure reasonable performance in most regimes. Very low *f* values ($f<0.5$) correspond to noticeable bias for the frequentist estimators. The initial estimator is largely robust to large skew in the allele frequency distribution, while the other two estimators are slightly biased by it at high levels of mixture. The data was simulated under the Balding–Nichols model as so the Bayesian method has an intrinsic advantage.

**Fig. 2:**
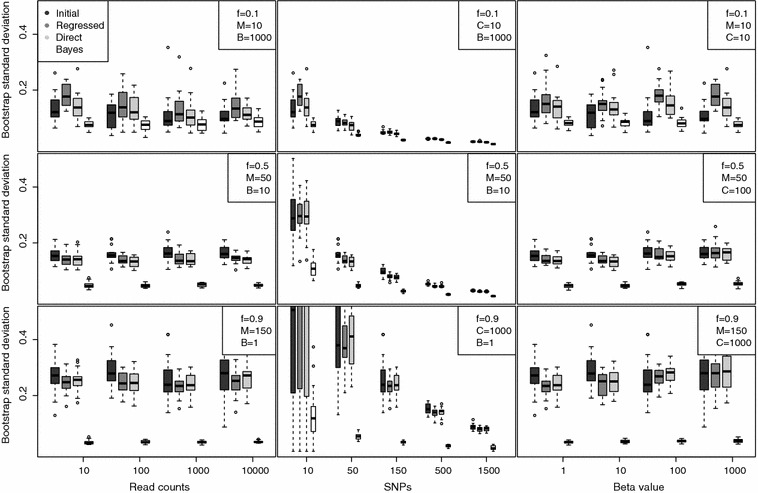
Boostrap standard deviation for each estimator for the same parameter values as Fig. 1. Specific simulated values can be found in Table 2. Each* boxplot* represents 50 bootstrap samples, each with 100 replicate data sets

Figure 2 shows that the estimator standard deviation was similar for the three frequentist estimators and markedly smaller in the Bayesian case. For each of the parameter regimes in Fig. 1, 100 bootstrap resamplings were performed. The standard deviation is largely diminished with increasing numbers of SNPs, with read counts and beta values playing little role. Note that bias for the frequentist estimators increases with increasing *f* values.

### Comparison in empirical data

**Fig. 3:**
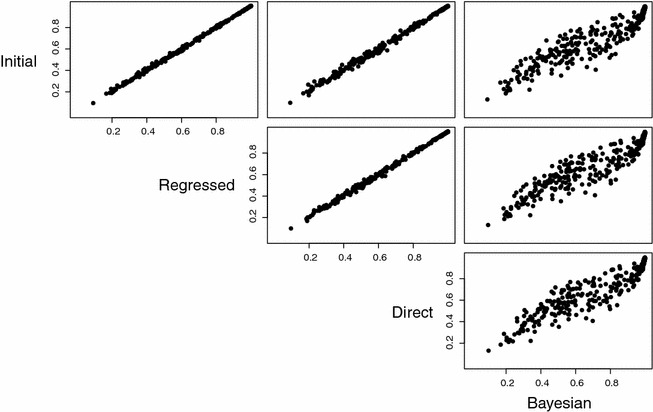
Correlation in inferred $f_i$ value for the four estimators across the set of 344 Ghanaian samples, with each sample represented as point. Each* panel* shows the correlation between the two estimators on the corresponding diagonal position. For the Bayesian case, the MAP value is reported

Since the underlying Balding-Nichols model within the simulations is likely misspecified relative to empircal data, performance was examined for each of the estimators applied to the WGS from 344 Ghanaian samples. The results shown in Fig. 3 show very strong correlation between the three frequentist estimators, with correlation better than 0.95. For the Bayesian estimate, the *maximum a posteriori* (MAP) estimate is reported. The Bayesian estimator is still highly correlated (>0.9) with the other estimators but is significiantly more variable in its estimates. Highly mixed and highly unmixed samples ($f\approx 0$ and $f \approx 1$) appear to have the most correlation, with moderately mixed samples deviating the most from the other three estimators.

### Comparison with COI

**Fig. 4:**
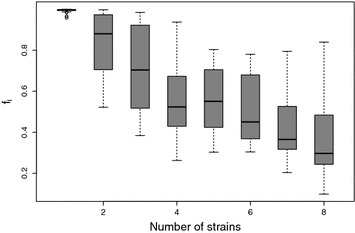
Boxplot of direct estimator $f_i$ for each of 344 Ghanian samples, grouped by number of inferred strains using the complex mixture model of [8]

As noted in the introduction, two recent efforts have extended MOI to WGS read count data and introduced the concept of COI [7, 8]. Both methods use finite mixture models to model the underlying number of strains in the sample. For comparison here, the model of O’Brien et al. is used, as it allows for more careful inference of the number of underlying strains and is more robust to errors in the read count data. For each of the 344 Ghanaian samples, the maximum *a posteriori* iteration is taken as the point estimate for the number of strains. Figure 4 graphs the relationship between the inferred number of strains and the F-statistic (a Spearman correlation of 0.83). While complex mixture models may provide a more penetrating understanding of within-sample variance, F-statistics appear to capture much of the same information in a single quantity.

### Laboratory strain data

The direct and Bayesian estimators were applied to the four clonal laboratory samples. The resulting values were very close to one, with the smallest observed value (0.98), above the standard threshold for clonality (0.95). The standard deviation of these estimates was less than 0.01. Within these samples, a moderate number of SNPs ($\sim$ 20) exhibited minor allele frequencies greater than 0.05, indicating some sequencing or alignment errors. These results indicate that these methods can reliably infer clonality even in the presence of some poorly-behaved SNPs.

### PF3K data set

**Fig. 5:**
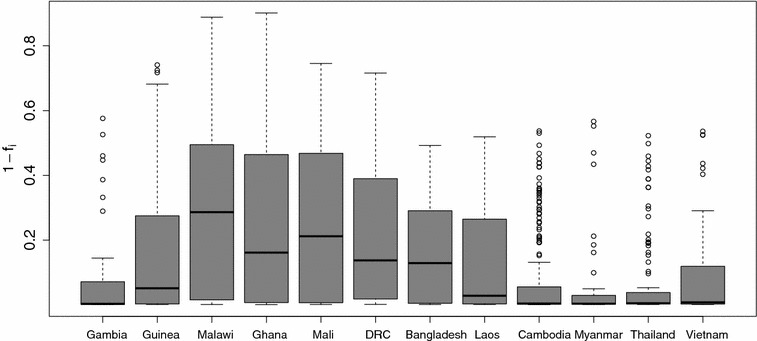
Boxplot of $1\,-\,f_i$ for each sample grouped by country of origin for 12 countries from the PF3K, arranged from west to east. The more intuitive $1\,-\,f_i$ is used to emphasize where low and high levels of mixture are prevelant

The PF3K clinical samples outlined in the Data section were grouped by country and used the direct estimator to calculate the inbreeding coefficient for each sample. These values are available on the companion website as a community resource [30]. Figure 5 summarizes the results, showing relatively low $f_i$ values throughout west Africa, with the noticeable exception of The Gambia. The median values of south and southeast Asian countries exhibit distinctly less mixture (higher $f_i$ values) than in West Africa. This is consistent with previous reports of highly variable amounts of within-sample mixture across countries [3]. Interestingly, while the median level of mixture mixture varies significantly across countries, highly mixed samples ($f_i<0.5$) and unmixed samples ($f_i>0.95$) are present everywhere.

These data overlap with two studies noted in the Background, where *f* values were also calculated using the initial estimator [17, 18]. Using the paper-reported values, we find that there is a 0.97 correlation with the samples from Ghana, and 0.96 for those from Guinea and the Gambia, against the direct estimator values found here. The high correlation between these estimates highlights the similar properties of the initial and direct estimators and indicates the strong consistency of these estimates across different data cleaning procedures.

## Discussion

This work presents several new approaches to inferring inbreeding coefficients using read counts from WGS, including a frequentist estimator that is significantly simpler and more intuitive than the initial estimator as well as a Bayesian approach that derives from a classical population genetics model. These approaches help connect MOI investigations to a broader set of work within population genetics and conservation ecology that may be helpful in control efforts [31]. This work also demonstrates a strong correlation between these metrics and the results of more complex mixture models for inferring COI [7, 8]. While not intended to supplant these more involved methods for investigating the within-sample mixture, this additional tool can assist researchers in connecting *P. falciparum* population genetics to a larger literature. To assist other researchers, the implementaton of these methods is also provided as an R package, pfmix, with tutorials and example datasets in an open-source framework at the package site, along with the direct estimates for the PF3K data set[30].

The model underlying the inbreeding coefficient makes a number of assumptions about the structure of the read count data and the biological mixing process that may affect inference. For the read count data, read counts are assumed to be unbiased and the SNPs are unlinked. While short read data can be biased in several ways, previous research indicates that mixture proportions calculated by read count ratios are largely unbiased (for instance, see [3] supporting information). However, *P. falciparum* exhibits significant linkage disequilibrium on scales significantly larger than the average distance between neighbouring SNPs in the data. This violation is not expected to bias the estimates as this absence of independence occurs (roughly) evenly across the genome. However, inference from a small region of the genome will likely exhibit bias.

A perhaps more troublesome assumption is embedded in the underlying structure of the *F*-statistic. An *F*-statistic measures the departure of the observed number of heterozygotes relative to those expected under Hardy–Weinberg equilibrium. In the context of mixed *P. falciparum* infections, the equilibrium assumptions—random mating, no selection, large population size, genetic isolation—are likely each violated at some level. For example, the mixture within a sample may be the result of a small number of founding individuals or be strongly selected by the human immune system. Without a more general approach to understanding the mixing process, anticipating the robustness of these estimates to this sort of misspecification is difficult. However, we do find that the PF3K samples from Cambodia that possess quite significant population structure still exhibit strong correlation between $f_w$ and the inferred number of strains.

As genomic data enables more elaborate statistical models for mixed infections and a broader understanding of *P. falciparum* genetic epidemiology, it will still be useful for field researchers to connect their work with population genetics and ecology through simple metrics. These issues are also relevant for researchers in a number of other *Plasmodium* species and protozoa with similar life-cycles. Inbreeding coefficients, which have a history going back to the beginnings of modern genetics, connect to a number of population genetic quantities such as effective population size and genetic drift [9, 32, 33] and may serve to complement traditional MOI values and newer models to this end. This work meets this need by providing a basis to infer these quantities and a suite of open-source tools for researchers to calculate them.

## Abbreviations

WGS: whole-genome sequence data
MOI: multiplicity of infection
COI: complexity of infection
HWE: Hardy–Weinberg equilibrium
SNP: single nucleotide polymorphism
PF3K: *Plasmodium falciparum* 3000 genomes
